# Coating of magnetite with mercapto modified rice hull ash silica in a one-pot process

**DOI:** 10.1186/2193-1801-3-515

**Published:** 2014-09-11

**Authors:** Nuryono Nuryono, Nur Mutia Rosiati, Bambang Rusdiarso, Satya Candra Wibawa Sakti, Shunitz Tanaka

**Affiliations:** Department of Chemistry, Faculty of Mathematics and Natural Sciences, Universitas Gadjah Mada, Yogyakarta, 55281 Indonesia; Division of Environmental Science Development, Graduate School of Environmental Earth Science, Hokkaido University, Hokkaido, Japan; Division of Environmental Material Science, Graduate School of Environmental Earth Science, Hokkaido University, Hokkaido, Japan

**Keywords:** Magnetite, Silica, Rice, Coating, One-pot process

## Abstract

In this research, mercapto-silica coated magnetite (Fe_3_O_4_-SiO_2_-SH) has been prepared in aqueous solution through a simple approach so called a one-pot process. The Fe_3_O_4_-SiO_2_-SH was prepared in nitrogen condition by mixing magnetite, 3-mercaptopropyltrimethoxysilane (MPTMS), and sodium silicate (Na_2_SiO_3_) solution extracted from rice hull ash, and adjusting the pH of 7.0 using hydrochloric acid. The residue was washed with deionized water, dried at 150°C and separated with an external magnetic field. In that work, the volume of MPTMS and Na_2_SiO_3_ was varied and the total amount of Si represented as silica was kept constant. Characters of the material including the functional group presence, the structure, the porosity, the morphology and stability toward various solvents were identified and evaluated. Results of characterization indicated that mercapto-silica has been coated magnetite particle with a simple one-pot process. Coating mercapto-silica on magnetite increases particle size, surface area, and chemical stability. Additionally, Fe_3_O_4_-SiO_2_-SH also shows high stability toward various organic solvents. The magnetic property of magnetite does not change after coating and the addition of nonmagnetic material still gives high value of maximum saturation magnetization. The presence of mercapto groups effective for interaction with heavy metal ions, the high chemical stability without removing the magnetic property promises the prospective application of Fe_3_O_4_-SiO_2_-SH in the future such as for separation and removal of heavy metal ions from aquatic environments.

## Introduction

Among the magnetic materials, iron oxides play a major role in many areas of chemistry, physics and material sciences. In particular, magnetic iron oxides such as magnetite (Fe_3_O_4_) and maghemite (γ-Fe_2_O_3_) have been investigated intensively for environmental and bio-applications (Daniel-da-Silva et al. 2007, 2008; Rebolledo et al. 2008; Hong et al. 2008; Li et al. 2007). Magnetite based materials with high magnetic characteristic are very effective as adsorbents for heavy metal ions removal. Superparamagnetic particles adhered to the target can be removed very quickly from a matrix using a magnetic field, but they do not retain their magnetic properties when the field is removed (Yantasee et al. 2007). However, it should be pointed out that uncoated magnetic nanoparticles are highly susceptible to oxidation when exposed to atmosphere and also susceptible to leaching under acidic conditions (Ren et al. 2008; Mahmoudi et al. 2011). In addition to convenient magnetic properties and low toxicity and price, Fe_3_O_4_ exhibit high surface to volume ratios, depending on the particle size, which associated to their ability for surface chemical modification can enhance the capacity for heavy metal adsorption in water treatment processes.

Inorganic polymers, such as silica, have been used as stabilizing agents for iron oxide and the silica coating has attractive properties including high biocompatibility (Mahmoudi et al. 2011), adsorption capacity, acid–base properties, insolubility in most solvents, and chemical and thermal stability (Yang 2003). In addition, silica can be grafted with a variety of functional groups, leading to considerable enhancement of their surface properties. Surface modification achieved by the attachment of inorganic shells or/and organic molecules not only stabilizes the nanoparticles, eventually preventing their oxidation, but also provides specific functionalities that can be selective for ion uptake. This system also has several advantages compared with conventional and other adsorbents in that the process does not generate secondary waste and the materials involved can be recycled and facilely used on an industrial scale. Furthermore, the magnetic particles can be tailored to fix and separate metal species in water, wastes, or slurries (Ren et al. 2008; Ngomsik et al. 2006; Li et al. 2008; Hu et al. 2005; Yavuz et al. 2006; Hai et al. 2005; Chang and Chen 2005; Liu et al. 2008; Zhou et al. 2009).

Modification of silica coated magnetite may be carried out into two steps namely coating silica on magnetite and functionalization on the silica coated magnetite (Lin et al. 2011; Shishehbore et al. 2011). The latter step is conducted by reacting silanol groups on the silica surfaces with organic compounds contaning silane groups at a high temperatur and in water free solvent to prevent hydrogen bonding that may marker the silanol groups. Nowaday, that non-green process has to be avoid. As the silica sources, organosilane agents such as tetraethoxyorthosilane (TEOS) are normally used (Yang et al. 2009; Pang et al. 2012). Using this precursor, two steps (hydrolysis and condensation) are involved in silica gel formation. However, using sodium silicate solution as the precursor, addition of acid to the solution results in the silica gel formation without hydrolysis step as reported by Lin et al. (2011). Sodium silicate that may be produced by treating rice hull ash with sodium hydroxide has been reported as precursor for preparation of ionic imprinting amino modified silica (Sakti et al. 2013), and sulfonato modified silica (Azmiyawati et al. 2012; Sulastri et al. 2011). Rice hull ash is a solid waste of agricultural products potential as row material for preparation of new silica based materials due high content of silica (80–90%) (Sakti et al. 2013).

This paper reports a simple and green approach of coating magnetite with mercapto modified silica in aqueous solution using sodium silicate solution made of rice hull ash as the precursor and 3-mercaptopropyltrimethoxysilane as the mercapto group source. Additionally, chemical reactions that occur in the aqueous solution during coating process are proposed, and the effect of coating on the magnetite properties and stability toward various types of solvents are evaluated.

## Materials and methods

### Materials

Chemicals used included FeCl_2_.4H_2_O, FeCl_3_.6H_2_O, HCl 37%, and NH_4_OH 25% supplied from Merck as receipted without any prior treatment for preparation of magnetite (Fe_3_O_4_), and commercial Fe_3_O_4_ from Aldrich used for a control material. For coating the magnetite was used Na_2_SiO_3_ solution (13% SiO_2_) produced from treatment of rice hull ash and mercaptopropyltrimethoxysilane (MPTMS) from Merck.

### Synthesis of magnetite

Magnetite, Fe_3_O_4_, was prepared using a simple chemical co-precipitation method reported anywhere. Typically, 2.0 g of FeCl_2_ · 4H_2_O and 5.2 g of FeCl_3_ · 6H_2_O were dissolved in 1 mL aqueous HCl (37%). The FeCl_2_ · 4H_2_O and FeCl_3_ · 6H_2_O aqueous solution was then added rapidly with 200 mL of deionized water and the solution was continuously sonificated under nitrogen for 1 h. Upon adding an aqueous NH_4_OH solution (25%, 15 mL), a distinctive black precipitate of Fe_3_O_4_ was formed immediately and the precipitate was kept overnight in a room temperature. The Fe_3_O_4_ was isolated and purified by centrifugation and then washed with water three to four times to remove excess NH_4_OH solution. The magnetite resulted was dried in an oven at 80°C for 2 h. The analog work was carried out with mixing technique of mechanical stirring.

### Coating magnetite with mercapto modified silica

About 0.5 g of freshly prepared Fe_3_O_4_ dispersed in 1 mL of HCl solution was added with certain volume of sodium silicate solution, MPTMS and deionized water; hence the total volume of the solution was 6 mL. The mixture then was added with solution of HCl 1 M or NH_4_OH 1 M drop wise to reach the pH of 7.0. The resulting precipitate was separated with an external magnet, washed with deionized water and dried at 80°C for 2 h. The mole ratio of SiO_2_ in sodium silicate solution to MPTMS was varied as presented in Table 1.

**Table 1:** **Variation of the coating material amount**

Product	Volume/amount of coating agents	
	Na _2_SiO _3_ (mL/mole SiO _2_)	MPTMS (mL/mole)
Fe_3_O_4_-SH	0.0/0.0	1.27/6.8
Fe_3_O_4_- SiO_2_-SH (25:75)	1.5/1.7	0.95/5.1
Fe_3_O_4_- SiO_2_-SH (50:50)	3.0/3.4	0.63/3.4
Fe_3_O_4_- SiO_2_-SH (75:25)	4.5/5.1	0.32/1.7
Fe_3_O_4_- SiO_2_	6.0/6.8	0.00/0.0

### Characterization of products

#### Characterization with fourier transform infrared (FT-IR) spectrophotometry

About 0.5 mg of product was homogenized with 200 mg of KBr powder and was converted into a pellet form with 2000 psi in pressure. The pellet was put in a sample cell and the absorbance was measured at a wave number range of 300–4000 cm^-1^.

#### Elemental analysis

The content of elements (C, H, N) was determined with a Yanaco CHN CORDER MT-6 Elemental Analyzer, and a Dionex Ion Chromatography was used to analyze the content of sulfur.

#### Identification of structure with X-ray diffraction (XRD)

In this characterization, the sample was grounded and put in a sample cell and analyzed with XRD. Cu k_α_ radiation from 40 kV and 30 mA was applied to the sample with a 2θ range of 5–70° (*scan speed of* 5°/min).

#### Identification of morphological products

The morphologies of all products were examined using a transmission electron microscopy (TEM) (JEM 1400) with 120 kV power, and frame size 1024 × 1024 pixel.

#### Porosity analysis

The Brunauer-Emmett-Teller (BET) surface area analysis was conducted using the nitrogen adsorption-desorption method (GSA. type NOVA 1200) with degassing temperature of 300°C for 3 h.

#### Measurement of magnetization values

The magnetization values of the products were identified using vibrating sample magnetometer (Oxford) at the maximum external magnetic field of 1.2 Tesla at 25°C.

#### Evaluation of product stability toward acid and organic solvents

Resulted product (0.5 g) was mixed with 15 mL of HCl solution 1 M, shaked for 5 min and kept in a room temperature. From the mixture, 1 mL of the substrate was collected in one day interval for 5 days, and analyzed the content of dissolved iron with atomic absorption spectrophotometry. Additional work was carried out to examine the stability of the product by dispersing 10 mg of coated silica in 50 mL various solvents and stirring the dispersion for 1 min.

## Results and discussion

### Effect of mixing techniques on magnetite character

Addition of NH_4_OH solution to a mixture of Fe^2+^ and Fe^3+^ solutions leads to form back magnetite precipitate. The mole ratio of Fe^2+^to Fe^3+^ 2:1 is needed to fulfill the stoichiometric reaction as presented in Eq. (1).
1

Oxidation of Fe^2+^ to Fe^3+^ in magnetite may occur easily in the presence of oxygen; hence, free oxygen condition by flowing nitrogen may prevent oxidation reaction as expressed in Eq. (2).
2

One of factors influencing the characters of magnetite is stirring technique during co-precipitation process. In this work, the synthesis of magnetite was carried out with two stirring techniques namely mechanical and ultrasonic ones. The yield of magnetite materials synthesized through both stirring techniques is presented in Table 2. From Table 2 can be seen that ultrasonic technique gives the yield percentage of magnetite higher than the mechanic one due to effectiveness of interaction between Fe^2+^/Fe^3+^ with OH^-^ to form hydroxides.

**Table 2:** **Yield of magnetite synthesized with two stirring techniques**

Mixing Technique	Yield	
	(g)	(%)
Mechanic	2.19	94.4
Ultrasonic	2.30	99.1

**Figure 1:**
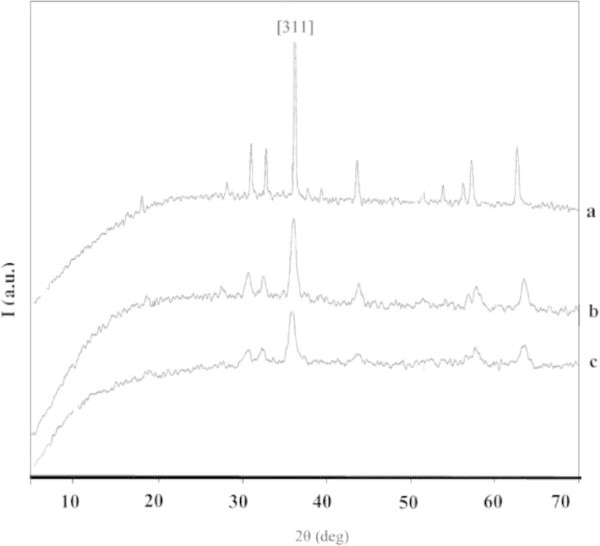
**XRD patterns of (a) commercial magnetite, (b) magnetite synthesized with ultrasonic and (c) mechanical techniques.**

The success of magnetite synthesis may be shown from the XRD pattern (Figure 1), in which the peaks of synthesized Fe_3_O_4_ appear at the similar 2θ to those of commercial one. In the commercial magnetite (c-Fe_3_O_4_) is observed a highest peak at 35.48° of space index [311]. This index peak also appears in magnetite synthesized through both mechanical (m- Fe_3_O_4_) and ultrasonic (u-Fe_3_O_4_) techniques. However, the peak intensity indicating the crystalline level is different with the order of c-Fe_3_O_4_ > u-Fe_3_O_4_ > m-Fe_3_O_4_. Evaluation of index peak [311] at 2θ ~ 35.5, several parameters of crystal including interspaces distance (d), lattice parameter (a), peak intensity (I) and average crystallite diameter () have been calculated and presented in Table 3.

**Table 3:** **Parameters of magnetite calculated from XRD pattern**

Magnetite	2θ(°)	Intensity	a (Å)	d _311_(Å)	(nm)
c-Fe_3_O_4_	35.48	957	8.21	2.53	28.93
m-Fe_3_O_4_	35.55	185	8.41	2.52	14.84
u-Fe_3_O_4_	35.62	274	8.31	2.52	14.21

Table 3 shows that m-Fe_3_O_4_ gives peak intensity lower and the crystallite size represented by  smaller than u-Fe_3_O_4_. Energy of ultrasonic wave improves effectiveness of stirring, homogenous dispersion and results in the small size crystallite. Higher stirring rate means larger energy transfer to crystallite and resulting in smaller sizes. Yang et al. (2009) reported that single phase formation of magnetite with sonochemical technique takes time only 1 h and 16 h is needed if mechanical technique is applied.

In conclusion, the use of ultrasonic wave energy probable may improve homogeneity of the mixture and effectiveness of contact between reactant particles to form precipitate of Fe(II)/Fe(III) hydroxide, as well as crystalline structure. Therefore, magnetite synthesized with ultrasonic technique was used for the further experiments.

### Characters of mercapto-silica coated magnetite

Coating of magnetite with silica and mercapto groups was conducted in aqueous solution. It involved a gelation process of silica followed with attachment of mercapto groups from MPTMS through one sol–gel reaction in the same process. The method was chosen since it offers benefits such as organic solvent free, simple and occurs at a room temperature. Functionalization of mercapto groups on silica modified magnetite at higher temperature may decompose MPTMS as the mercapto source before attachment of the functional groups on the silica surface occurs. In this work, coating was carried out by varying the amount ratio of sodium silicate to MPTMS at constant weight of magnetite and the results can be seen in Table 4.

**Table 4:** **Yield of coated magnetite materials before and after washing with deionized water and fraction of coated material**

Product	Weight (g)		Weight fraction of coated material (%)
	Before washing	After washing	
Fe_3_O_4_- SiO_2_	1.84	0.70	28.57
Fe_3_O_4_- SiO_2_-SH (75/25)	1.96	0.95	47.37
Fe_3_O_4_- SiO_2_-SH (50/50)	1.52	0.91	45.05
Fe_3_O_4_- SiO_2_-SH (25/75)	1.43	1.00	50.00
Fe_3_O_4_-SH	1.17	1.17	57.27

Table 4 shows that washing decreases the weight of products, except for Fe_3_O_4_-SH where sodium silicate solution was not used. Sodium chloride may be produced from reaction between sodium silicate and HCl. This byproduct may be trapped in the coated magnetite as impurity. Washing with deionized water may dissolve that salt and may improve the purity of the coated magnetite. Therefore, by using sodium silicate as the silica source, washing of the precipitate is important step to find the coated magnetite with higher purity. By assumption no magnetite loosing during coating, the weight fraction of silica and mercapto group can be calculated and presented in the last column of Table 4.

#### Elemental analysis

Analysis of three elements was carried out to support indication of the success of coating mercapto-silica on magnetite and the result is expressed in Table 5. It is observed that hydrogen is detected in magnetite and silica coated magnetite samples due to presence of water hydrated or silanol in the samples. Coating mercapto modified silica on magnetite is revealed by the presence of sulfur from thiol groups in both materials of magnetite coated with mercapto modified silica. The weight percentage ratio of S to C (S/C) obtained (0.77 for Fe_3_O_4_-SiO_2_-SH(50:50) and 0.99 for Fe_3_O_4_-SiO_2_-SH) is comparable to the value of S/C for MPTMS calculated theoretically (0.89). The component fraction represented as weight percentage of merapto-silica coated magnetite may be calculated based on the yield weight and elemental content of the samples. Fe_3_O_4_-SiO_2_-SH(50:50) consists of 54.05% Fe_3_O_4_, 30.87% SiO_2_ and 15.08% mercaptopropyl groups. This result shows higher content of mercapto groups in comparison to the previous data (2.57% S) reported by Zhang et al. (2013). Additionally, calculation shows that Fe_3_O_4_-SiO_2_-SH(50:50) and Fe_3_O_4_-SH contain mercapto groups of 1.31 and 4.11 mmol/g sample, respectively.

**Table 5:** **Result of elemental analysis in magnetite samples**

Sample	Content of element (%)		
	C	H	S
u-Fe_3_O_4_	n.d	0.41	n.d
Fe_3_O_4_-SiO_2_	n.d	0.42	n.d
Fe_3_O_4_-SiO_2_-SH (50:50)	5.49	1.38	4.20
Fe_3_O_4_-SH	13.21	2.64	13.15

n.d: non detected.

Coating mercapto-silica on magnetite results in the larger product weight and the increase is in-line with the amount of MPTMS added (Table 4). With constant weight of magnetite, the capability in mole to bond silicone from both sodium silicate and MPTMS is also constant. Since the molecular weight of MPTMS is larger than that of silica, inclining the MPTMS weight causes the increase of coated magnetite weight.

Before being coated, magnetite was acidified to form the active sites on the magnetite surface facilitating the interaction between the magnetite surface and reagent to be coated. Silica coated magnetite was carried out by adding 1 M HCl solution or 1 M NH_4_OH solution drop wise on a mixture of sodium silicate and magnetite to reach the pH of 7.0, and insoluble gel is formed. At pH 7.0, silicate anion from sodium silicate solution may form siloxane bonds. At low pH (acidic condition) magnetite can be dissolved and the silica formed was converted from SiO_2_ to Si(OH)_4_. At higher pH (alkaline) the siloxane bonds are broken to produce silicate anions (Kalapathy et al. 2002).

**Figure 2:**
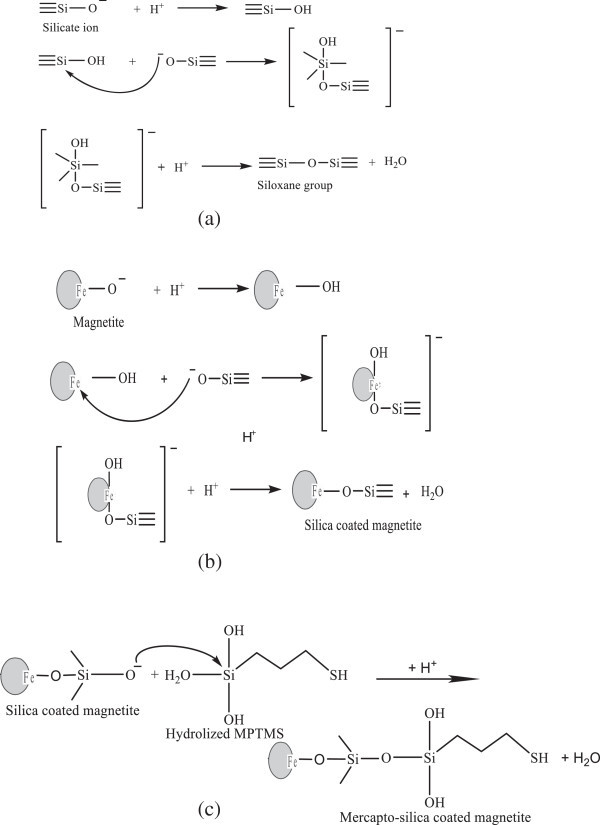
**Formation model of Fe**_**3**_ **O**_**4**_ **-SiO**_**2**_ **-SH from sodium silicate solution; (a) formation of Si-O-Si, (b) formation of Fe-O-Si on magnetite surface, and (c) attachment of mercapto-propyl group on silica coated magnetite.**

Different from TEOS or TMOS, the use of sodium silicate as the precursor does not involve hydrolysis process. By adding acid, a part of silicate anions is protonated to form silicate acid driving force to form siloxane bonding with another silicate anion. Siloxane bond formation leads to the oligomerization reaction (formation of silica network). Therefore, gel formation occurs due to condensation of silicate anion and acid, and the reaction may be expressed in Figure 2(a) (Sulastri et al. 2011).

Addition of HCl in coating magnetite leads to protonation of oxygen atom on magnetite giving to lower negative charge density on Fe atom. It makes easily to be attracted by electron pair of siloxy group (Si-O^-^) from silicate anion to form Fe-O-Si group. The reaction of attachment of silica on magnetite can be modeled in Figure 2(b) (Durdureanu-Angheluta et al. 2008).

The presence of silane agent from MPTMS may involve the formation of siloxane bonding with silica bonded to magnetite and it leads to attachment of mercapto modified silica on magnetite. Attachment of mercaptopropyl groups on silica is initiated by hydrolysis of methoxy groups from MPTMS in basic solution to form silanol (Si-OH) groups and these groups may react with silicate anion coated on magnetite to form Fe-O-Si-O-Si-(CH_2_)_3_-SH (Figure 2(c)).

#### Functional groups of coated magnetite

**Figure 3:**
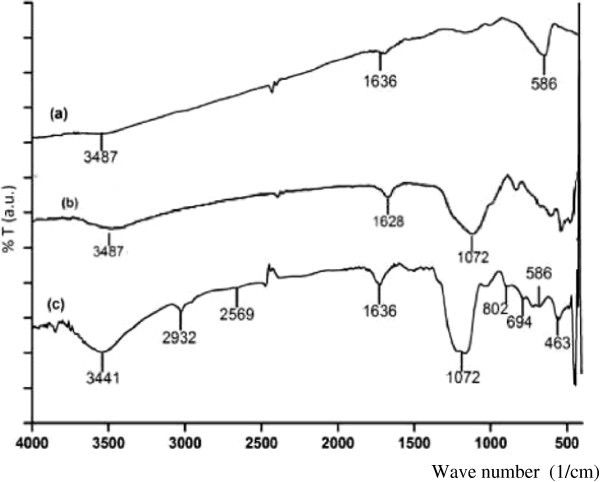
**Infrared spectra of (a) u-Fe**_**3**_ **O**_**4**_ **, (b) Fe**_**3**_ **O**_**4**_ **-SiO**_**2**_ **, and (c) Fe**_**3**_ **O**_**4**_ **-SiO**_**2**_ **-SH (50:50).**

Analysis with infrared spectroscopy is aimed to identify the change of functional group presence due to coating of magnetite. IR spectra resulted from the measurement for three different magnetite materials is presented in Figure 3.

In FT-IR spectra of magnetite (Figure 3(a)) is observed an absorbance band at 586 cm^-1^ corresponding Fe-O bond and it is attributed to formation of ferrite phase. Different from magnetite, FT-IR spectra of silica coated magnetite, Fe_3_O_4_-SiO_2_, (Figure 3(b)) shows pronounce changes, particularly at region of 1300–700 cm^-1^, indicating the presence of silica coating. The presence of silica coated on magnetite is shown with characteristic band at 463 cm^-1^ from bending vibration of Si-O-Si. Asymmetric bending vibration corresponding to Si-O-Si bonding is revealed with absorbance band at 1072 cm^-1^. Absorbance bands in coated magnetite FT-IR spectra around 1628–1636 cm^-1^ and 3441–3487 cm^-1^ come from bending and stretching vibration, respectively, of –OH groups from both Fe-OH and Si-OH. Stretching vibration of Si-O-H bonding results in an absorbance band at 960 cm^-1^. In FT-IR spectra of magnetite coated only with silica, absorbance of Si-OH vibration does not appear clearly due to overlap with broad band of stretching vibration from Si-O-Si.

In comparison to magnetite and silica coated magnetite, FT-IR spectra of mercapto-silica coated magnetite, Fe_3_O_4_-SiO_2_-SH(50:50), (Figure 3(c)) gives characteristic absorbance of propyl and mercapto groups from MPTMS. The C-H bonding of propyl groups results in absorbance at 2932 cm^-1^ corresponding to bending and asymmetric vibration of C-H. The presence of mercapto groups is identified by the appearance of bands at 694 and 879 cm^-1^ that can be assigned to asymmetric stretching of C-S and bending vibration of S-H. Weak bands at 2569 cm^-1^ in IR spectra of mercapto coated magnetite is additional proof of the presence of -SH groups.

#### Structure of coated magnetite

**Figure 4:**
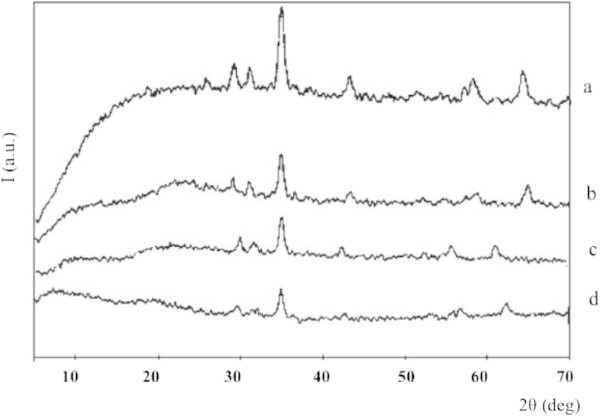
**XRD patterns of magnetite materials; (a) u-Fe**_**3**_ **O**_**4**_ **, (b) Fe**_**3**_ **O**_**4**_ **-SiO**_**2**_ **, (c) Fe**_**3**_ **O**_**4**_ **-SiO**_**2**_ **-SH (50:50) (c) and (d) Fe**_**3**_ **O**_**4**_ **-SH.**

To effect of the sodium silicate to MPTMS mole ratio on the structure of the coated magnetite resulted, the coated magnetite samples were analyzed with XRD method and the result is expressed in Figure 4. It can be seen from Figure 4 that coating process does not change the peak position but the intensity, particularly for index [311], decreases with increasing the amount of MPTMS added. It means that coating process does not lead to change the crystallite structure but the presence of the mineral but reduces level. Addition of amorphous materials such as silica and MPTMS may cover the crystallite and lead to the peak intensity lower. The change of intensity and D_XRD_ quantitatively has been calculated and presented in Table 6.

**Table 6:** **Parameters of coated magnetite materials calculated from XRD pattern**

Material	2θ(°)	Intensity	(nm)
u-Fe_3_O_4_	35.62	274	14.21
Fe_3_O_4_-SiO_2_	35.43	126	25.12
Fe_3_O_4_-SiO_2_-SH (50:50)	35.43	115	27.25
Fe_3_O_4_-SH	35.19	80	21.06

Table 6 reveals larger crystallite size of coated magnetite in comparison to that of without coating one indicating that coating on the magnetite surface is proved. Magnetite coated only silica (Fe_3_O_4_-SiO_2_) gives bigger crystallite size than that coated only MPTMS (Fe_3_O_4_-SH). It is probable due to the oligomerization of silica before coating on the magnetite surface. In contrast, the presence of mercapto propyl groups in MPTMS inhibits oligomerization reaction and result in the formation of thin one layer MPTMS coated magnetite. Combination coating of silica and MPTMS gives biggest crystallite size among the investigated samples. Oligomerization of silica combined with attachment of MPTMS on the silica is suspected as the factor affecting the crystallite size.

#### Morphology of coated magnetite

**Figure 5:**
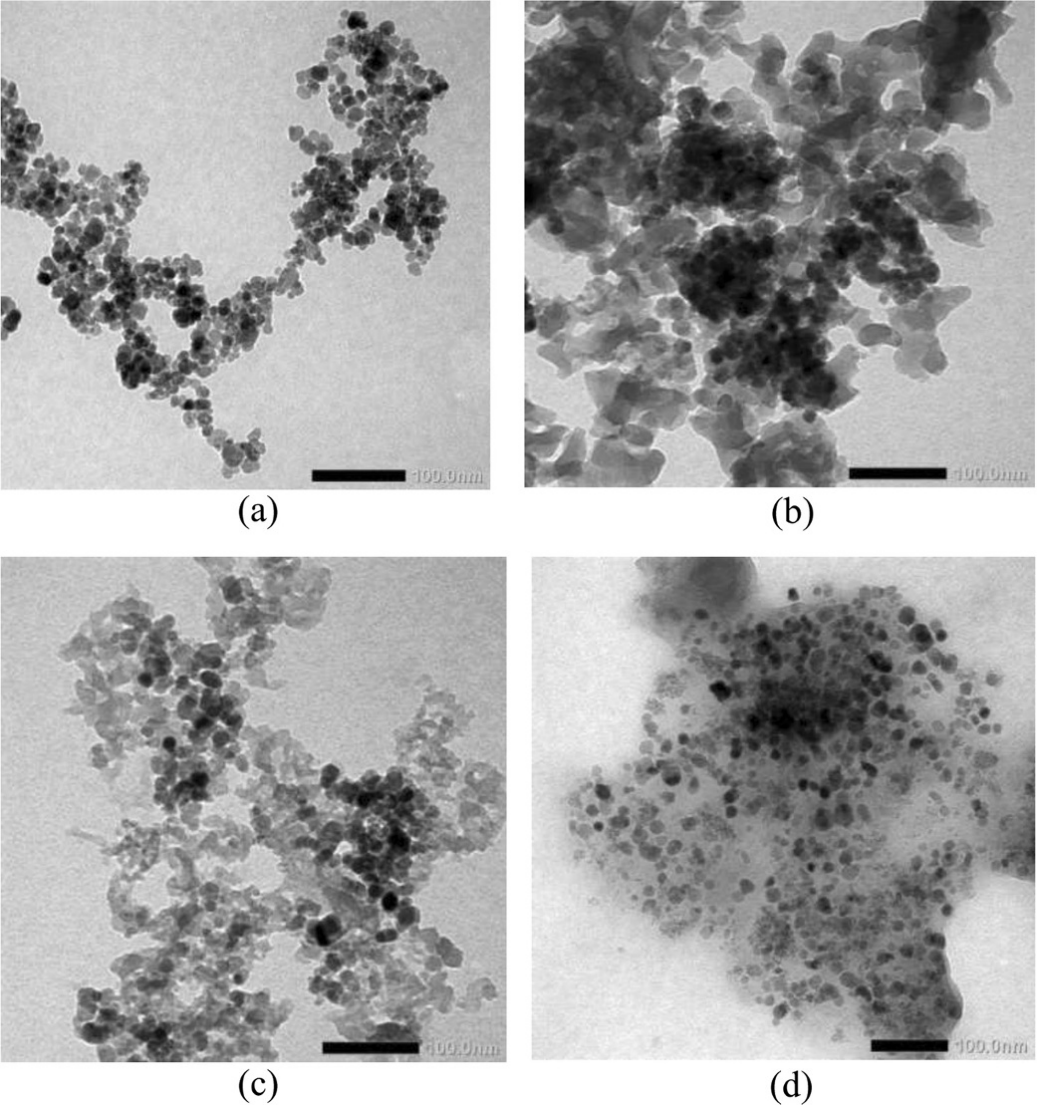
**TEM image of magnetite materials; (a) u-Fe**_**3**_ **O**_**4**_ **, (b) Fe**_**3**_ **O**_**4**_ **-SiO**_**2**_ **, (c) Fe**_**3**_ **O**_**4**_ **-SiO**_**2**_ **-SH(50:50), and (d) Fe**_**3**_ **O**_**4**_ **-SH.**

**Figure 6:**
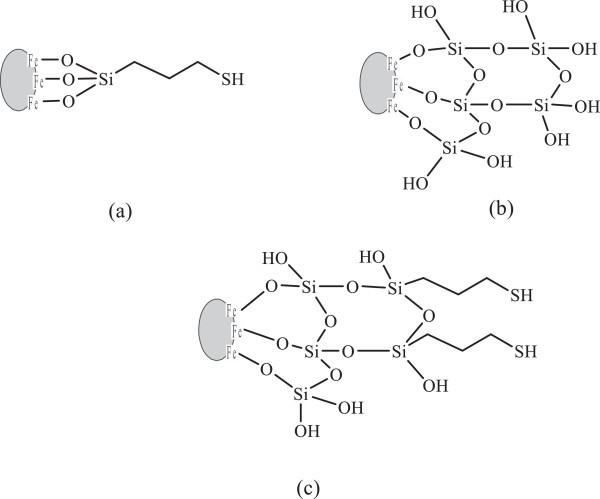
**Structural model of magnetite materials; (a) Fe**_**3**_ **O**_**4**_ **- SH, (b) Fe**_**3**_ **O**_**4**_ **- SiO**_**2**_ **, and (c) Fe**_**3**_ **O**_**4**_ **-SiO**_**2**_ **-SH (50:50).**

Morphology of magnetite was identified with transmission electron microscope (TEM) and the result is expressed in Figure 5. In Figure 5(a) is a TEM image of magnetite without coating that can be seen the presence of small black and gray spherical particles of magnetite. From the TEM image of mercapto and silica coated magnetite (Figure 5(b-d)) is showed that there are aggregates of bigger particles consisting of small back sphere as magnetite seed that are surrounded by gray shell of silica. This is another proof of the coating success. Due to the aggregate formation, however, particle size is difficult to be estimated. However, qualitatively may be estimated from Figure 5 that the order of particle size is u-Fe_3_O_4_ > Fe_3_O_4_-SiO_2_-SH (50:50) > Fe_3_O_4_-SiO_2_ > Fe_3_O_4_-SH. Identification results support the reaction modelled in Figure 2 and the hypothetic structure of coated magnetite materials can be expressed in Figure 6.

#### Porosity

Another important character of magnetite is particle porosity including surface area and pore size that may be measured using BET analysis. The result of analysis is summarized in Table 7 revealing that surface area of coated magnetite increases with increasing particle size (Table 6). As described before, silica coated on magnetite may experience oligomerization reaction and exhibit porous layers outside of magnetite seed. In contrary magnetite coated only with MPTMS gives smallest surface area due to only one layer of MPTMS formed on the surface of magnetite. On magnetite coated with silica and MPTMS have the greatest particle size as well as highest surface area. Reaction between silica with MPTMS results in thicker coating on magnetite. Based on Table 7, the porous diameter average of all coated magnetite materials is higher than 20 Å that can be categorized as mesoporous material.

**Table 7:** **BET Data**

Material	Surface area (m ^2^/g)	Porous total volume (cm ^3^/g)	Porous diameter average (Å)
Fe_3_O_4_-SiO_2_	82.24	0.42	101.09
Fe_3_O_4_-SiO_2_-SH(50:50)	162.52	0.48	58.74
Fe_3_O_4_-SH	3.42	0.02	108.94

#### Magnetic property of coated magnetite

**Figure 7:**
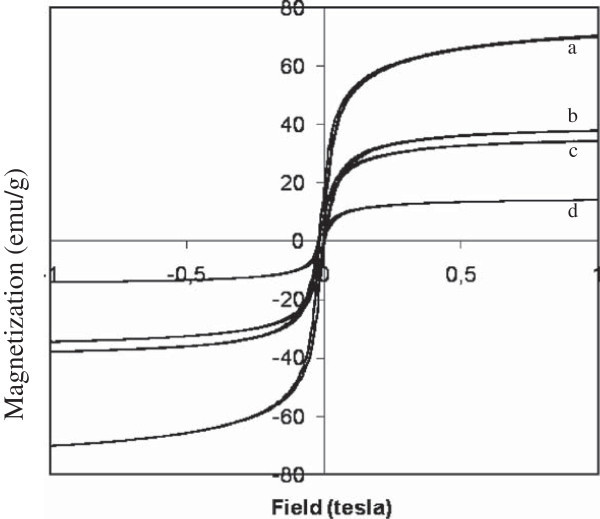
**VSM hystheresis curve of magnetite materials; (a) u-Fe**_**3**_ **O**_**4**_ **, (b) Fe**_**3**_ **O**_**4**_ **-SH, (c) Fe**_**3**_ **O**_**4**_ **-SiO**_**2**_ **, and (d) Fe**_**3**_ **O**_**4**_ **-SiO**_**2**_ **-SH(50:50).**

The effect of magnetite coating on magnetic property may be evaluated from the data of analysis. This analysis utilizes a power of the external magnetic field to produce a hysteresis curve describing the magnetic properties of samples. This curve resulted from analysis of coated magnetite is expressed in Figure 7, and magnetic parameters including saturation field value (M_s_), coercivity field (H_c_) and permanent magnetization (M_r_) can be calculated and presented in Table 6. The hysteresis curve represents the energy required for magnetization. From Figure 7 seems that all magnetite (coated and un-coated) give small curve area indicating low energy for magnetization and are classified as soft magnet. This assumption is supported by the low H_c_ values of the samples. The value of H_c_ ≠ 0 for uncoated and coated magnetites indicates ferrimagnetic properties of the samples. Similar results that coating decreases the energy of magnetization have been reported by previous researchers. Zhang et al. (2013) reported that coating magnetite with the same material (thiol-silica) reduces the maximum saturation magnetization from 55.05 to 20.14 emu/g. Coating magnetite with amino-silica material leads to decrease of magnetization (Lin et al. 2011). It was explained that this decrease was ascribed to the contribution of the nonmagnetic NH_2_/SiO_2_ layer to the total mass of the particles.

From Table 8, based on the M_s_ value, can be seen that coated magnetite gives lower magnetic property than without coated one. Magneticity of single layer coating (silica or MPTMS) is higher than that of double layer coating (silica and MPTMS). This phenomenon is consistent with the crystallite size in Table 6. The increase of particle size leads to declining the magnetic property. Large particle size results in low mobility and is difficult to be attracted by external magnetic field.

**Table 8:** **Magnetic parameters of magnetites**

Material	M _s_ (emu/g)	H _c_ (×10 ^-2^ Tesla)	M _r_ (emu/g)
u-Fe_3_O_4_	70.39	1.78	16.28
Fe_3_O_4_-SiO_2_	34.26	1.85	7.89
Fe_3_O_4_-SiO_2_-SH (50:50)	14.05	1.15	3.27
Fe_3_O_4_-SH	37.75	1.85	7.43

#### Stability of coated magnetite toward various solvents

**Figure 8:**
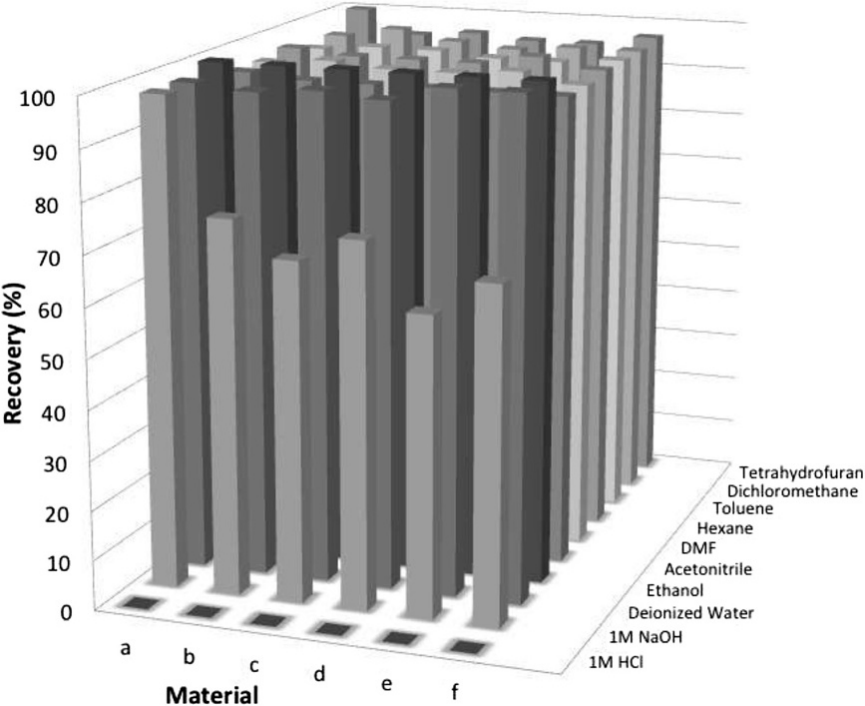
**Recovery of (a) u-Fe**_**3**_ **O**_**4**_ **, (b) Fe**_**3**_ **O**_**4**_ **-SiO**_**2**_ **, (c) Fe**_**3**_ **O**_**4**_ **- SiO**_**2**_ **-SH(75:25), (d) Fe**_**3**_ **O**_**4**_ **-SiO**_**2**_ **-SH(50: 50), (e) Fe**_**3**_ **O**_**4**_ **-SiO**_**2**_ **-SH(25:75) and (f) Fe**_**3**_ **O**_**4**_ **-SH by magnet after 10 times dispersion-recollection from various solvents.**

One of objectives of coating is to protect magnetite from chemical reaction. In this work, we evaluated the effect of coating on the chemical stability of magnetite toward various solvents. Magnetite sample was mixed with various solvents for certain period and collected by using external magnet. Several solvents such as nonpolar solvents (hexane and toluene), polar aprotic solvents (dichloromethane and tetrahydrofurane) and polar solvents (deionized water and ethanol) were used in this experiment. In Figure 8 is shown that almost all of tested samples could be recovered from various types of solvents with different polarity even after 10 cycles of dispersion and recollection. There is no significant mass lost after recollection. It indicates that magnetite based materials resulted are relatively stable toward various solvents with different polarity. However, the different phenomena was observed when the magnetite materials were mixed with strong acidic or basic solution. As can be seen in Figure 8, all magnetite materials tested are not recollected at all using an external magnet when those are dissolved in 1 M HCl solution. In acidic solution magnetite is dissolved to form Fe^2+^/Fe^3+^ions whereas undissolved silica based material that is not attracted with magnet and remains in the solution as suspension. However, the addition of 1 M NaOH leads to weight loss partly of the tested samples. It may be understood since NaOH solution is able to dissolve silica based silica to form silicate ion but not for magnetite which is stable in basic condition and recollected with an external magnet.

**Figure 9:**
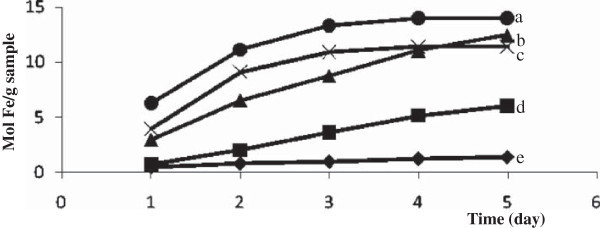
**The amount of Fe dissolved in 2 M HCl within 5 days from magnetite materials; (a) Fe**_**3**_ **O**_**4**_ **-SiO**_**2**_ **-SH(25:75), (b) Fe**_**3**_ **O**_**4**_ **- SiO**_**2**_ **-SH(75:25), (c) Fe**_**3**_ **O**_**4**_ **-SiO**_**2**_ **-SH(50: 50), (d) Fe**_**3**_ **O**_**4**_ **-SiO**_**2**_ **and (e) Fe**_**3**_ **O**_**4**_ **-SH.**

**Figure 10:**
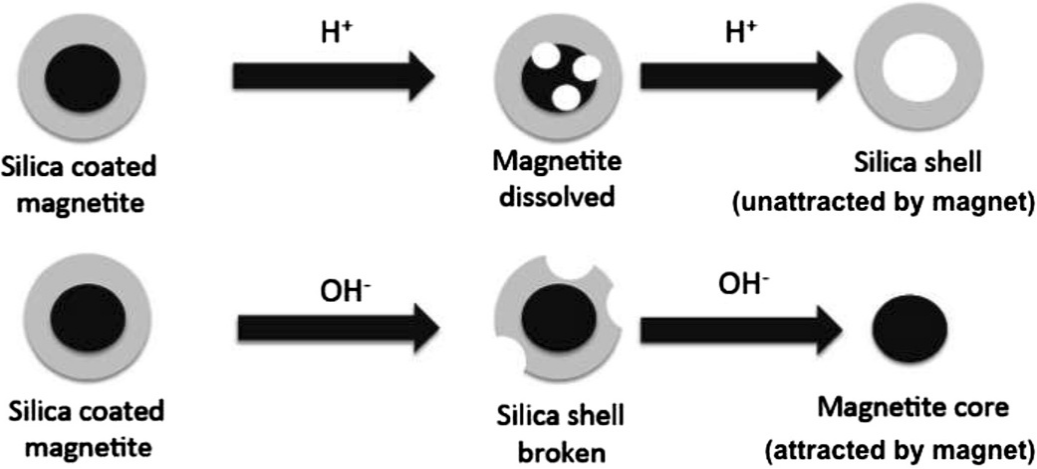
**Dissolution model of silica coated magnetite material in acid solution and in base solution.**

Additionally, the chemical stability was also evaluated based on the amount of Fe released to the solution after the magnetite materials were dispersed in 2 M HCl solution for 5 days and the result is presented in Figure 9. In comparison to other types of magnetites, Figure 9 shows that Fe_3_O_4_-SH is the most stable among the examined magnetites toward HCl solution. According to Lam et al. (2008), mercaptopropyl groups from MPTMS tend to not interact with proton; hence this coating is able to protect magnetite from acid. It is different from magnetite coated with silica where oxygen atom of silica may interact with proton that leads to weaken the siloxane bonding. Magnetite with double coating tends to have lower stability than that with single coating. It has not been understood the reason, however, probable due to the surface area effect. Magnetite with double coating (Fe_3_O_4_-SiO_2_-SH) gives largest surface area (Table 7) so that effective to contact to proton and enhance the solubility of magnetite. Siloxane bond of silica on magnetite surface may react with OH^-^ to form silicate, which is soluble in water. Further interaction of silica and OH^-^ breaks and dissolve silica coated and leaving magnetite core. Scheme of reaction between silica coated magnetite with acid or base is illustrated in Figure 10.

## Conclusions

In this research, synthesis of mercapto-silica coated magnetite using sodium silicate solution prepared from rice hull ash as the silica source has been developed in aqueous solution through a simple and facile preparation approach called one pot process. This approach is rapid and does not require the addition of any surfactant to form mercapto-silica hybrid coated directly to magnetite. The presence of mercapto-silica on magnetite not only improves the stability of magnetite but also gives high potency as active sites effectively for heavy and hazardous metal ions. Material of magnetite coated with mercapto modified silica still shows magnetic property and can be attracted with external magnetic field. Therefore, it is expected that in the future mercapto-silica coated magnetite may be promoted as prospective adsorbent for simple separation of heavy metal ions from industrial waste water.
